# Function and evolution of *Magnaporthe oryzae* avirulence gene *AvrPib* responding to the rice blast resistance gene *Pib*

**DOI:** 10.1038/srep11642

**Published:** 2015-06-25

**Authors:** Shulin Zhang, Ling Wang, Weihuai Wu, Liyun He, Xianfeng Yang, Qinghua Pan

**Affiliations:** 1 State Key Laboratory for Conservation and Utilization of Subtropical Agrobioresurces, and Guangdong Provincial Key Laboratory for Microbe Signals and Crop Disease Control, South China Agricultural University, Guangzhou, 510642, China; 2 Hainan Key Laboratory for Monitoring and Control of Tropical Agricultural pests, Environment and Plant Protection Institute, Chinese Academy of Tropical Agricultural Sciences, Haikou, Hainan 571101, China

## Abstract

*Magnaporthe oryzae* (*Mo*) is the causative pathogen of the damaging disease rice blast. The effector gene *AvrPib*, which confers avirulence to host carrying resistance gene *Pib*, was isolated via map-based cloning. The gene encodes a 75-residue protein, which includes a signal peptide. Phenotyping and genotyping of 60 isolates from each of five geographically distinct *Mo* populations revealed that the frequency of virulent isolates, as well as the sequence diversity within the *AvrPib* gene increased from a low level in the far northeastern region of China to a much higher one in the southern region, indicating a process of host-driven selection. Resequencing of the *AvrPib*allele harbored by a set of 108 diverse isolates revealed that there were four pathoways, transposable element (TE) insertion (frequency 81.7%), segmental deletion (11.1%), complete absence (6.7%), and point mutation (0.6%), leading to loss of the avirulence function. The lack of any TE insertion in a sample of non-rice infecting *Mo*isolates suggested that it occurred after the host specialization of *Mo*. Both the deletions and the functional point mutation were confined to the signal peptide. The reconstruction of 16 alleles confirmed seven functional nucleotide polymorphisms for the *AvrPib*alleles, which generated three distinct expression profiles.

Plant pathogens have evolved a number of so-called “pathogenicity” genes, which act to allow them to successfully infect their host. Functionally, their products are concerned with either the formation of the infection structure, the control of turgor pressure or the production of toxins^1^,^2^. A second group of genes determines the ability of the pathogen to infect a specific host plant^3^,^4^,^5^. The basis of pathogen race / host genotype compatibility lies most commonly in a gene-for-gene interaction, in which the product of a host disease resistance (*R*) gene senses the presence of a corresponding pathogen avirulence (*Avr*) gene^4^,^6^,^7^. A successful R/Avr interaction results in the activation of the host’s defense response, thereby suppressing the infection^7^,^8^,^9^,^10^,^11^. Understanding the mechanistic basis of these gene-for-gene interactions is seen as a key means of developing durable genetics-based strategies for the management of plant disease.

The blast pathogen *Magnaporthe oryzae* (*Mo*) is the holomorph of a complex of heterothallic Ascomycetes, and is able to infect more than 50 different grass species. The interfertile anamorph *Pyricularia oryzae* is responsible for blast in both rice and some other species^12^,^13^. Blast is one of the most damaging of rice diseases, and – although to date only in South America- the disease has the potential to threaten wheat production as well^5^,^14^. The rice blast pathosystem has emerged as an effective model for studying gene-for-gene interactions^3^,^14^. Of the >100 recognized major host *R* genes, 22 have been successfully isolated to date^7^,^15^,^16^,^17^,^18^, while the same is true for eight of the >40 *Avr* genes known^5^,^16^,^19^,^20^. In contrast to the host gene products, which are almost members of the NBS-LRR family of proteins, there is little evidence of any commonality among the *Avr* products^4^,^15^,^16^,^21^. As a result, the R/Avr interaction appears to rely on a diversity of molecular processes^5^,^7^,^13^, a situation which will only be rationalized when a larger set of *Avr* sequences becomes available. The present research targeted the rice *R* gene *Pib*, which evolved in the *indica* gene pool. The gene confers a high level of resistance to most Japanese *Mo* isolates^22^ and has protected much of the rice cropped in NE China for some years^23^,^24^. The paper describes the map-based cloning of *AvrPib*, and the application of population genetic, comparative genomic and mutagenic approaches to characterize its function and evolution.

## Results

### Genetic and physical mapping of *AvrPib*

**Figure 1:**
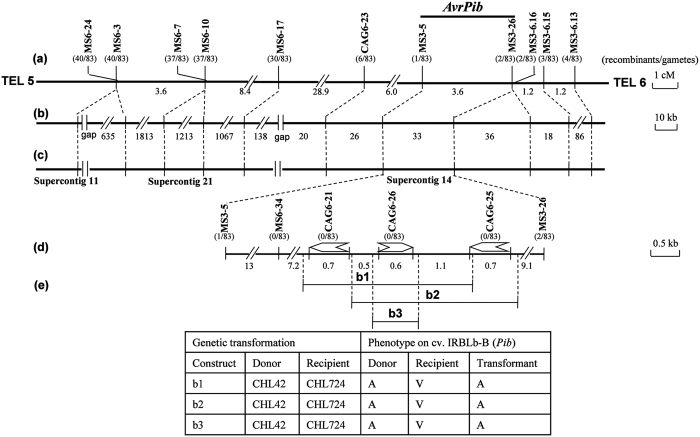
Map-based cloning of *AvrPib*. (**a**) Localized genetic map around *AvrPib*. The numbers shown below the map indicate genetic distances between adjacent markers in cM, and the number of recombinants between each marker is indicated. (**b**) Localized physical map, showing the separation in kb between adjacent markers. (**c**) Localized contig map of the *AvrPib* region in the reference *Mo* isolate 70-15. (**d**) A fine-scale physical map reveals three CAGs as candidates for *AvrPib*. (**e**) Complementation test of candidate genes. The disease reaction of transgenic isolates on IRBLb-B plants is shown as either A (avirulent) or V (virulent).

Isolate CHL42 was avirulent and CHL381 virulent when used to infect IRBLb-B, and both isolates were virulent on cv. LTH. The 83 single ascospore progeny isolates derived from the CHL42 x CHL381 cross segregated as 41 avirulent on IRBLb-B and 42 virulent, consistent with the presence of a single avirulence gene (χ^2^ (1:1) = 0.012, *P *> 0.99), designated *AvrPib*. The bulk segregant analysis showed that ten of the 121 informative SSR markers on chromosome 6 (MS6-24, 6–3, 6–7, 6–10, 6–17), and chromosome 3 (3–5, 3–26, 3–6.16, 3–6.15 and 3–6.13) (Supplementary Table S1) each discriminated between the DNA bulks, indicating that the *AvrPib* locus embodying chromosome was rearranged by compositing of both the chromosomes. A subsequent linkage analysis (Fig. 1a) revealed 40 recombinants between *AvrPib* and MS6-24, but only four between *AvrPib* and MS3-6.13. The closest linked of the chromosome 3 markers were MS3-5 (one recombinant with *AvrPib*), MS3-6.16 and MS3-26 (two recombinants) and MS3-6.15 (three recombinants), indicating that the *AvrPib* locus lay in the 3.6 cM segment flanked by MS3-5 and MS3-26 on chromosome 3. The physical length of the equivalent segment in the reference isolate 70-15 is 33 kb (Fig. 1b,c). Four candidate avirulence gene (CAG) markers (CAG6-21, 6–23, 6–25 and 6–26), along with one further SSR marker (MS6-34) - all lying within the MS3-5 to MS3-26 segment - were then used to genotype the 40 recombinants between *AvrPib* and MS6-24 and the four between *AvrPib* and MS3-6.13. Four of these five markers (the exception was CAG6-23 which was rearranged into a region between markers MS6-17 and MS3-5) co-segregated perfectly with *AvrPib* (Fig. 1d). Each of the three linked CAG markers thus represented a possible candidate for *AvrPib*, so was subjected to a genetic complementation assay.

### Genetic complementation of candidate genes for *AvrPib*

The two constructs b1 (harboring CAG6-21 and 6–26) and b2 (CAG6-26 and 6–25) were transformed separately into isolate CHL724. Since both constructs converted the isolate’s virulence to avirulence, it was concluded that CAG6-26 was the most likely candidate for *AvrPib* (Fig. 1e). As confirmation, the introduction of the single transgene construct b3 (harboring just CAG6-26) was also shown to confer the avirulence phenotype. The CAG6-26 coding sequence (CDS) represented in isolate CHL42 was identical to that of the locus present in isolate 70-15, whereas the CHL42 CAG6-21 and CAG6-25 CDSs both differed (Supplementary Fig. S1). The conclusion was that CAG6-26 is *AvrPib*, and that isolate 70-15 is avirulent on *Pib* because it too carries *AvrPib*.

### Population structure of *AvrPib*

**Figure 2:**
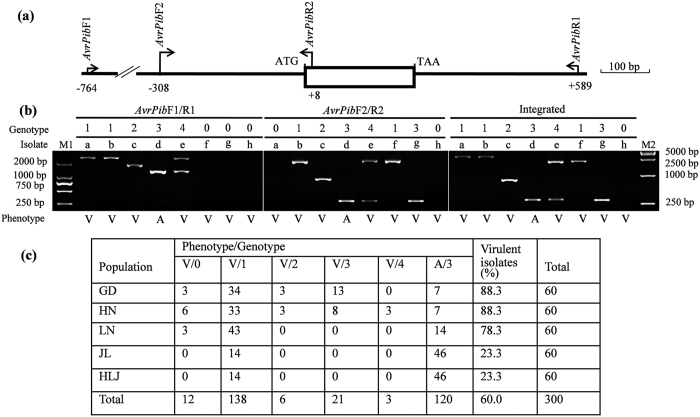
Allelic variation for *AvrPib*. (**a**) The two primer pairs *AvrPib*F1/R1 and *AvrPib*F2/R2 detect the presence of the *Pot2* and *Pot3* transposons in the 5’ region of the gene. The CDS is represented by open box. (**b**) Typing of eight *Mo* isolates. The *AvrPib*F1/R1 and *AvrPib*F2/R2 amplicons were used to distinguish five alleles: 0 (no amplification), 1–3 (one fragment of variable size) and 4 (two fragments); the disease reaction of each isolate when inoculated on IRBLb-B was either A (avirulent) or V (virulent). The different genotypes assigned in a given isolate were integrated by positive ones. The phenotype/genotype is given to each isolate as: a, CHL2600 (V/1), b, CHL2626 (V/1), c, EHL0348 (V/2), d, CHL2345 (A/3), e, EHL0342 V/4), f, CHL2549 (V/1), g, CHL2417 (V/3), h, EHL0370 (V/0); M1, M2: size markers. (**c**) Allelic variation among the five *Mo* populations collected in Guangdong (GD) and Hunan (HN) in South, and Liaoning (LN), Jilin (JL), and Heilongjiang (HLJ) in Northeast China (also see Supplementary Table S2).

The presence of *AvrPib* was tested by infecting IRBLb-B plants with representative isolates from five *Mo* populations (each comprising 60 monoconidial isolates), collected from the southern Chinese provinces Guangdong (GD) and Hunan (HN), the near northeastern province Liaoning (LN) and the far northeastern provinces Jilin (JL) and Heilongjiang (HLJ). Virulence was much more frequent in the southern populations (88.3% in both GD and HN) than in the far northeastern ones (23.3% in both JL and HLJ). The virulence frequency in LN was 78.3%. The indication was therefore that positive selection on the *AvrPib* was steadily increased from far northeastern to southern regions. Based on the amplicons generated by two primer pairs (Fig. 2a,b), five distinct genotypes were recognized (designated 0 through 4). In combination with the phenotype, six states were identified: virulence + genotype 0 (V/0), V/1, V/2, V/3, V/4 and A/3 (Supplementary Table S2). V/1 and A/3 were the commonest isolate types, with the others restricted to the southern populations. Two types (V/1 and A/3) were detected in JL and HLJ, three (V/0, V/1, and A/3) in LN, five in GD (V/0, V/1, V/2, V/3 and A/3) and all six in HN (V/0, V/1, V/2, V/3, V/4 and A/3). These results indicated that genetic diversity of *AvrPib* was steadily increased from far northeastern to southern regions.

### Phylogeny and allelic variation of *AvrPib* alleles

**Figure 3:**
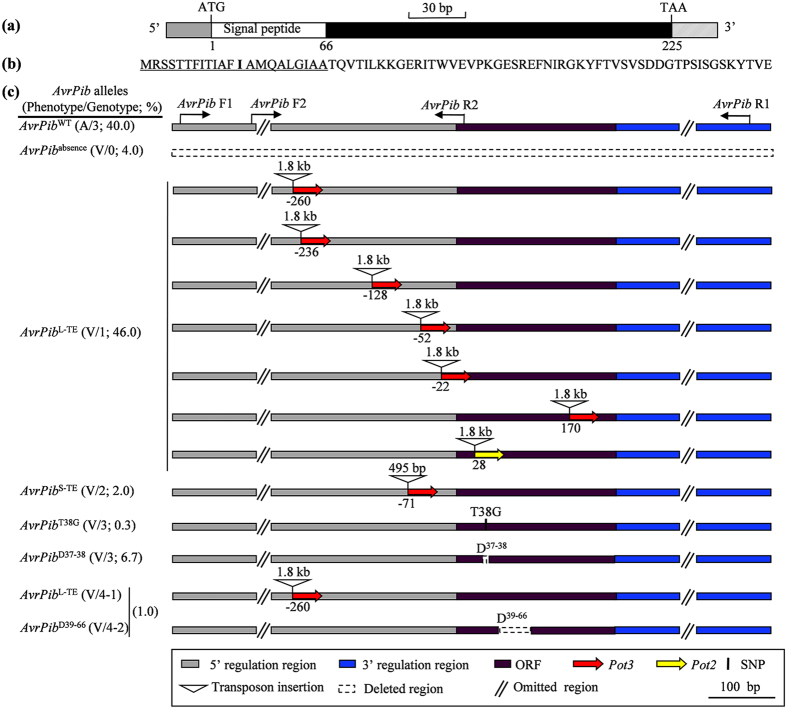
Characterization of allelic variation at *AvrPib*. (**a**) The structure of the gene. (**b**) The predicted AvrPib polypeptide sequence; the signal peptide sequence is shown underlined and the bold letter is a hot variable site. (**c**) The functional nucleotide polymorphic maps of the seven natural alleles in the five *Mo* populations. WT, wild type; L-TE, a larger TE insertion; S-TE, a smaller TE insertion; D^37–38^, deletion at positions 37 and 38; D^39–66^, deletion at positions 39 to 66. The primers used to generate sequencing templates are indicated. Phenotype/genotype designations were shown in Fig. 2 and frequency of each phenotype/genotype was counted based on Supplementary Table S2.

A phylogenetic analysis of the *AvrPib* sequence was based on 111 sequences. (Although only 108 isolates were sampled, three of the genotype 4 isolates (EHL0317, EHL0342, EHL0394) were found to harbor two sequences (Table S2, Fig. S2).) The isolate 70-15 *AvrPib* sequence was also included. The analysis identified two large groups (designated A and B), corresponding to genotypes 1 and 3, respectively. Genotype 2 isolates fell into group B, while the genotype 4 ones were distributed across both groups. These results indicated that the *AvrPib* alleles were certainly grouped into two large groups, as per the presence/absence of transposon element (TE) insertion. The standard *AvrPib* allele (as for example the one present in isolate CHL42, designated *AvrPib*^WT^) was an intronless 225 bp sequence, encoding a 75 residue polypeptide, the N-terminal 22 residues of which formed a predicted signal peptide (SP) (Fig. 3a,b). A survey of 300 isolates identified seven putatively functional nucleotide polymorphisms (FNPs) for the natural *AvrPib* alleles (Fig. 3c; Supplementary Table S2). The largest group (46.0% of all 300 isolates, designated *AvrPib*^L-TE^) harbored a larger TE insertion in both the 5’ and CDS regions, while the *AvrPib*^WT^ allele was present in 40.0% (Fig. 3c). The remaining five groups were present at only a low frequency. They comprised (1) *AvrPib*^D37–38^, featuring a deletion at positions 37 and 38 (6.7%), (2) *AvrPib*^absence^, a complete absence of *AvrPib* (4.0%), (3) *AvrPib*^S-TE^, featuring a smaller TE insertion in the 5’ region (2.0%), (4) *AvrPib*^D39–66^, harboring a deletion from positions 39 to 66 (1.0%) and (5) *AvrPib*^T38G^, featuring a single base change mutation at position 38 (0.3%). To address the question as to whether TE insertion was driven by host selection, an additional set of 12 *Mo* isolates, adapted to Gramineae species other than rice, was analyzed (Supplementary Table S2). None of these isolates harbored a TE insertion. Taken together, there were four pathways, TE insertion (including the natural alleles *AvrPib*^L-TE^ and *AvrPib*^S-TE^), deletion (*AvrPib*^D37–38^ and *AvrPib*^D39–66^), absence (*AvrPib*^absence^), and point mutation (*AvrPib*^T38G^), leading to loss of the avirulence function of *AvrPib* in the five populations. Among them, the frequency of TE insertion was highest (81.7% of virulent isolates), followed by the deletion (11.1%), absence (6.7%), and the point mutation (0.6%) (Fig. 2; Supplementary Table S2).

### Validation of *AvrPib* alleles

**Figure 4:**
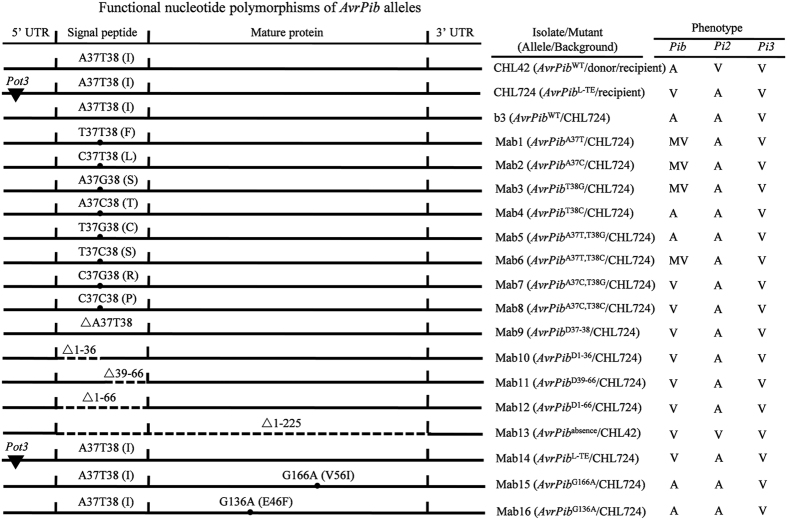
Validation of functional nucleotide polymorphisms for *AvrPib* alleles. Alleles generated by site-directed mutation, deletion and insertion indicated by “•”, “△”and “▼”, respectively. The sequences have not been drawn to scale. Deletions are shown by dotted lines. In the Mab13 knockout allele, the CDS was replaced by an *Hpt* cassette. Mab14 was constructed by domain swapping involving the *Pot3* transposon. The silent alleles Mab4, 5, 15 and 16 involved non-functional sequence variants. The phenotypes of each allele when challenged with *Pib, Pi2* and *Pi3* are given. A: avirulent, V: virulent, MV: moderately virulent.

**Figure 5:**
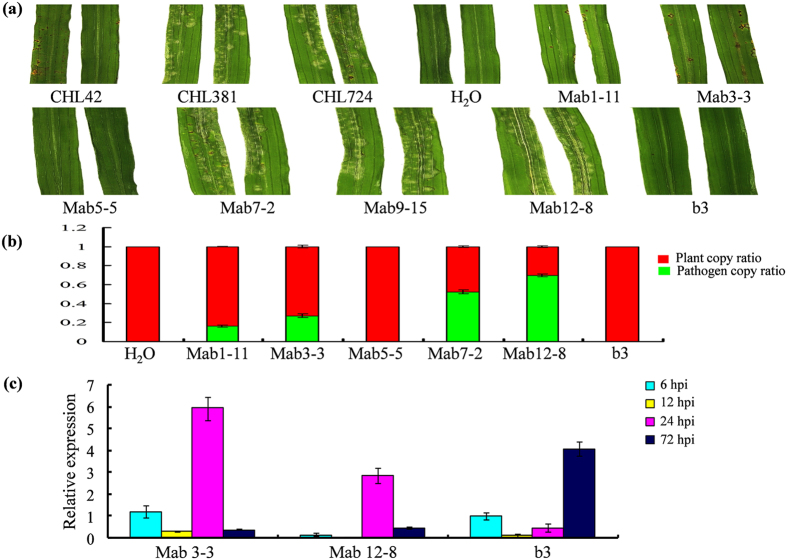
Expression profiles of the *AvrPib* alleles. (**a**) The disease response of IRBLb-B when inoculated with *Mo* strains carrying the various *AvrPib* variants. (**b**) A qPCR-based estimation of fungal abundance measured five days after inoculation. The quantification of plant and fungal DNA was inferred from the abundance of the rice *Ubi* and the *Mo Pot2* amplicons. (**c**) A qPCR-based estimation of *AvrPib* transcript abundance. The data shown in both (**b**) and (**c**) are given in the form mean ± standard deviation (*n *= 3). Similar results were obtained from two biological replicates each with three technical repeats. CHL42, CHL381: parental isolates used to create the mapping population, CHL724: the recipient isolate used for transformation with reconstructed alleles (these alleles are prefixed by “Mab”). H_2_O: mock inoculation control.

To assure the putative FNPs for *AvrPib*, the representative natural alleles were reconstructed. Because the majority of the deletions and the functional point mutation located at positions 37 and 38 in the SP, all possible *AvrPib* alleles (assigned as Mab1 to Mab9) focused on the two positions were created by site-directed mutagenesis (Supplementary Fig. S3a,b), and then tested for virulence against *Pib, Pi2* and *Pi3* (Fig. 4). Three phenotypes were recognized: both Mab4 (designated *AvrPib*^T38C^) and Mab5 (*AvrPib*^A37T,T38C^) were avirulent with respect to *Pib*, while Mab1 (*AvrPib*^A37T^), Mab2 (*AvrPib*^A37C^), Mab3 (*AvrPib*^T38G^) and Mab6 (*AvrPib*^A37T,T38C^) were all moderately virulent, and Mab7 (*AvrPib*^A37C,T38G^), Mab8 (*AvrPib*^A37C,T38C^) and Mab9 (*AvrPib*^D37–38^) were fully virulent (also see Fig. 5a). The three additional deletion mutants focused on the SP, Mab10 (*AvrPib*^D1-36^), Mab11 (*AvrPib*^D39–66^) and Mab12 (*AvrPib*^D1-66^), were also all virulent with respect to *Pib*. To reconstruct *AvrPib*^absence^ (Mab13), *AvrPib*^WT^ was knocked out by targeted gene replacement using an *Hpt* cassette (Figs S3c, d), while the *AvrPib*^L-TE^ (Mab14) was created by domain swapping with *Pot3* from the recipient isolate CHL724 to the donor isolate CHL42 (Supplementary Fig. S3e). Both Mab13 and Mab14 displayed virulence with respect to *Pib*. The significance of the single base mutations G166A (present in isolate CHL2417) and G136A (isolate EHL0329) (Table S2) was tested by creating *AvrPib*^G166A^ (Mab15) and *AvrPib*^G136A^ (Mab16). Both of these synthetic alleles displayed an avirulence reaction with respect to *Pib*. In light of these results, it was predicable that additional point mutations under the seven FNPs confirmed (Fig. 3) were all null mutations for *AvrPib*. All the reconstructed alleles showed the expected reactions as their recipients against *Pi2* and *Pi3* (Fig. 4).

### Expression profiles of *AvrPib* alleles

Since three phenotypes were observed among *AvrPib* alleles reconstructed (Figs 4,5a), two alleles of each phenotype were selected for quantification of their pathogenicities on the IRBLb-B via quantitative PCR (qPCR) assay. Pathogen growth was hardly detectable in host tissue infected by both avirulent isolates, Mab5-5 (*AvrPib*^A37T,T38G^) and b3 (*AvrPib*^WT^) (Fig. 5b). In contrast, the ratio between the copy numbers of *AvrPib* and the host gene *Ubi* reached 50% for the virulent mutant, Mab7-2 (*AvrPib*^A37C,T38G^) and 70% for Mab12-8 (*AvrPib*^D1-66^). As for the moderately virulent mutants, Mab1-11 (*AvrPib*^A37T^) and Mab3-3 (*AvrPib*^T38G^), the ratios were, respectively, 15% and 25% (Fig. 5b). To further characterize the dynamic expression profiles of the three different types of *AvrPib* alleles, one allele of each phenotype were selected for quantification of their transcription on the IRBLb-B via qRT-PCR assay. The level of transcription of avirulent isolate b3 fell over the period six to 12 hours post infection (hpi), and then rose again after 24 hpi, reaching a peak of >4 fold the background level by 72 hpi (Fig. 5c). The abundance of the moderately virulent Mab3-3 was similar that of b3 at both 6 and 12 hpi, but then was steady at six fold the background level between 24 and 72 hpi, after which it fell away. Finally, the virulent isolate Mab12-8 was transcribed at a lower level than either Mab3-3 or b3 at 6 hpi, and the level decreased further over the next six hours, reaching a three fold background level by 24 hpi before falling away.

## Discussion

The reverse genetics approach has successfully identified many pathogenicity genes^1^,^2^, but a few race/cultivar specificity genes in various pathosystems, indicating that the latter one was likely out of target for artificial mutation. Similarly for the host plant, few *R* genes have been identified from artificial mutants^15^. On the other hands, the forward genetic approach called map-based cloning has provided a highly robust means of identifying the genes involved in the plant/pathogen interaction^15^,^16^. The assumption is that each host *R* gene is matched by a pathogen *Avr* gene, although with respect to the rice/*Mo* pathosystem, the number of isolated *Avr* genes (eight) lags well behind that of the *R* genes (22), despite the fact that the size of the rice genome (*c*. 400 Mb) is an order of magnitude larger than that of *Mo* (*c*. 40 Mb). The small size of the pathogen genome tends to favor greater genomic plasticity, that are in turn powerful and fundamental counterforce against host selection pressure from the bigger cognates^25^,^26^,^27^,^28^,^29^. The resulting drastic divergences in micro- and macro-genomic regions that should be the major impediment, which can sometimes stop chromosome walking to the target locus in the map-based cloning process^19^,^30^,^31^,^32^. The complementary and/or alternative approaches applied in such cases were association genetics^33^, comparative genomics^20^, and comparative transcriptomics^32^, all of which rely on the high throughput capacity of current DNA sequencing technologies. In the current study, the *AvrPib* has been successfully isolated by map-based cloning, *in silico*, in which even the chromosome and its micro-genomic region containing the target locus was rearranged based on the recombinants detected among the polymorphic markers those were developed based on the reference sequence of isolate 70-15 (Fig. 1). That is, the rearrangement of targeted macro-/micro- genomic region (if any) is a crucial step for successful in map-based cloning of target gene, *in silico*, using the reference sequence.

Monitoring pathogen populations is an important input into elaborating strategies for the management of plant disease^14^,^34^,^35^,^36^. According to the gene-for-gene principle, the host *R* gene is a frequent and powerful driver of genetic structure and population dynamics of *Avr* gene^27^,^35^,^36^,^37^. It is clear that *Pib* has shaped the genetic architecture of *Mo* populations, since the clear geographical cline noted in the frequency of *AvrPib* mirrors the usage of this *R* gene in the Chinese rice crop (Fig. 2). Since both phenotypic and genotypic structures of the adjacent sub-regions within the northeastern region (LN *vs* both JL and HLJ), which is number one Great Plains in China, were largely different, indicating that host selection, but not geographic ones exerted on *AvrPib*. The restriction of the relatively rare V/2, V/3 and V/4 *AvrPib* types to *Mo* populations from southern China also reflects the action of host selection pressure rather than that of any geographic factor. *Pib*, introduced from International Rice Research Institute (IRRI) in early 1960s, was intensively used in *indica* rice breeding programs in southern China (especially in GD and HN provinces); it is carried by a number of successful cultivars, notably Teqing and Texianzhan25, which have been widely grown across southern China^38^,^39^. In contrast, the basis for the gene’s deployment in the northeastern part of the country can be probably be traced to just a single donor, the Japanese *japonica* cultivar BL1, introduced in the late 1970s, which has only given rise to a small number of cultivars grown over a rather limited area^40^,^41^. It was due to the tropical and subtropical ecosystems in southern China, which are similar with that in IRRI in the Philippines, is an *indica* rice adapted style, whereas the temperate ecosystem in northeastern China, which is similar with that in Japan, is a *japonica* rice specified style.

An understanding of the molecular basis of allelic variation in *Avr* genes can be exploited for elaborating a sustainable strategy of crop disease control^35^,^36^,^42^. The current genetic analysis of *AvrPib* has shown that its avirulence function has been lost most frequently via TE insertion, but deletion, absence and point mutation have also contributed (Fig. 2, Supplementary Table S2). Intriguingly, the deletions and the functional point mutation variants all involve the SP sequence (Fig. 4). This is not a general feature of *Avr* genes, however, since the same has not been observed with respect to the intensively researched *Avr* genes such as rice blast *AvrPik*^33^,^36^, and potato late blight *Avr3a*^43^ and *PiAvr2*^42^. Moreover, the global alleles of *AvrPib* reconstructed by artificial mutation in the SP region (Mab1 to 12; Fig. 4) showed three expression profiles in both phenotype and transcription (Fig. 5). Another particular feature of the allelic variation for *AvrPib* is that TE insertion has been such a predominant driver of the loss of avirulence. Considering most TE insertions were occurred in the 5’ region of *AvrPib* (Fig. 3), as well as the TE-inserted isolates all were with the null point mutations, if any (Supplementary Table S2), the implication is that the TE insertion that often serve as targets of selection, this may represent a cost effective way of modifying the native function of target gene – not by altering its product, but rather by abolishing its transcription^44^,^45^. Note that when a set of non-rice *Mo* isolates was investigated, none harbored a TE insertion in the *AvrPib* surrounding region (Supplementary Table S2), which was taken to indicate that the TE insertions in the rice *Mo* isolates was indeed driven by host selection. This, further, indicated that the TE insertion most likely occurred after the adaptation of *Mo* to domesticated rice.

## Methods

### Mapping population construction

The parents of the *Mo* mapping population were the hermaphroditic, *Pib*-avirulent isolate CHL42 (MAT1-2) and the male fertile, *Pib*-virulent isolate CHL381 (MAT1-1). Both isolates were collected from diseased rice plants growing in, respectively, Yunnan and Jiangsu provinces. Genetic cross and progeny isolation was performed as previously described^19^,^46^. To minimize the risk of both mutation and contamination, each isolate was archived using the filter paper-based method described elsewhere^19^. On the host side, the line IRBLb-B, which harbors *Pib* as its sole blast *R* gene, was bred from a cross between BL1 (*Pib* donor) and the highly blast susceptible cv. LTH^47^. Both IRBLb-B and LTH seedlings were inoculated with each of the parental and progeny isolates. Inoculation and the classification of phenotype assessment were performed^48^.

### Genetic and physical map construction

Genomic DNA of each *Mo* isolates was extracted from 200 mg mycelia using a Fungal DNA kit (Omega, Norcross, GA, U.S.A) following the manufacturer’s protocol. Two contrasting DNA bulks were prepared on the basis of the phenotypic outcome: one comprised DNA from eight avirulent isolates and the other from eight virulent ones. A total of 121 genome-wide simple sequence repeat (SSR) markers previously developed^49^ were applied to each of the two DNA bulks and the DNA of each of the two parental isolates. Based on the outcome of the bulk segregant analysis, the markers putatively linked to the *AvrPib* locus were used to generate a localized linkage map. The resolution of the map in the vicinity of the target locus was refined by adding an additional SSR marker and four CAGs (candidate avirulence genes)^19^,^46^. Genetic distances between a pair of adjacent marker loci is simply estimated by the ratio *r *= *N*_*r*_/*N*_*T*_, where *N*_*r*_ is the actual number of recombinants occurred in the distance, and *N*_*T*_ is the total number of the mapping population. A physical map surrounding the *AvrPib* locus was elaborated by locating the linked SSR sequences on the genomic sequence of the reference *Mo* isolate 70-15.

### Genetic complementation

The three sequences (b1 to b3), were amplified (primer sequences listed in Table S1) using Phusion High-Fidelity DNA polymerase (NEB, Beijing, China) from the genomic DNA of isolate CHL42. The amplicons were inserted into pMD20-T (TaKaRa, Dalian, China) to generate the three transgene constructs pMD20-T-b1 (harboring CAG6-21 and 6-26), b2 (CAG6-26 and 6-25) and b3 (CAG6-26). Following their amplification from the pMD20-T templates with primers incorporating an *Asc*I recognition site (Supplementary Table S1), the transgene sequences were introduced into the binary vector pBHT2-AscI. Prior to their transformation^50^ into isolate CHL724, each construct was first validated by sequencing. At least 14 independent hygromycin-resistant transformants per construct were carried forward for testing for virulence on the monogenic lines IRBLb-B (*Pib*), IRBLZ5-CA (*Pi2*) and IRBL3-CP4 (*Pi3*). Each inoculation experiment was conducted at least three times^48^.

### Genotyping, resequencing and phylogenetic analysis

Two primer pairs were designed, designated AvrPibF1/R1 and AvrPibF2/R2 (Supplementary Table S1). One pair targeted the full *AvrPib* sequence from position −764 to +589, and the other a 5’ segment from positions −308 to +8 (Fig. 2a). DNA extracted from field *Mo* isolates were amplified in two separate reactions, and given a genotype code of either 0 (no amplification with either primer pair), 1 (carrying the larger TE), 2 (carrying the smaller TE), 3 (lacking any TE) or 4 (two copies of *AvrPib*, one with larger TE and another without TE). A set of 12 *Mo* isolates adapted to barley and other grass species was tested with respect to the presence of TEs. The different genotypes assigned to a given isolate were integrated with priority on presence to absence of both *AvrPib* and TE (if any). A random set of 108 rice isolates, covering all of the types selected (a portion of the common A/3 and V/1 types and all representatives of the uncommon types) was chosen for re-sequencing purposes. The same segment present in five of the non-rice *Mo* isolates was also resequenced. Amplicons comprising a single fragment (genotypes 1 through 3) were directly sequenced, while the amplicon classed as genotype 4 was first cloned into pMD20-T to separate the two fragments present, which were then separately sequenced. The *AvrPib* allele sequences were aligned with the reference sequence of isolate 70-15 using DnaSP v5.0 software (http://www.ub.edu/dnasp/) to identify variant positions. The resulting 111 sequences (stretching from positions −710 to +530 of the reference sequence) were then subjected to a phylogenetic analysis using the bootstrap neighbor-joining method in conjunction with the Kimura two parameter model, implemented in MEGA 6 software^51^.

### Mutant reconstruction

To apply site-directed mutagenesis, primers harboring the target polymorphism were designed using a MutanBEST kit (TaKaRa, Dalian, China), according to the supplier’s protocol, then used to amplify from a pMD20-T-b3 template with Phusion High-Fidelity DNA polymerase. The products were inserted into pMD20-T for sequencing. The associated transgene constructs were obtained by treating with *Asc*I each of the amplicons produced by primer pair b3F/R (Supplementary Table S1), and the resulting products were then inserted into pBHT2-AscI. The *AvrPib*^absence^ allele was generated by targeted gene replacement. In brief, the 1.0 kb upstream (AvrPib-U) and 1.0 kb downstream (AvrPib-D) sequences of *AvrPib* were amplified from isolate CHL42 using primer pairs AvrPib-up-F/R and AvrPib-down-F/R, respectively (Table S1). The hygromycin phosphotranferase gene (*Hpt*) cassette (1.4 kb) was amplified from the plasmid pCX62^52^ using the primer pair AvrPib-Hpt-F/R (Table S1). AvrPib-U, AvrPib-D and *Hpt* were then introduced into pMD20-T to generate the plasmids T-AvrPib-U, T-AvrPib-D and T-*Hpt,* respectively. All three T-plasmids were ligated into pGKO2 to generate the cassette pGKO2-AvrPib-U*-Hpt-*AvrPib-D, which was then transformed into isolate CHL42. The gene replacement products were validated by PCR^53^ (Supplementary Fig. S3). *AvrPib* targeted domain swapping was performed to generate the *Pot3* insertion mutant. In brief, an amplicon harboring *Pot3* was produced from an isolate CHL724 template using the primer pair Mab14F/R (Supplementary Table S1). This PCR product was then fused to pMD20-T-b3 to generate pMD20-T-*Pot3*-b3 by restriction ligation based on *Hind*III and *Bst*BI digestion. The pMD20-T-*Pot3*-b3 construct was finally inserted into pBHT2-AscI for transformation as described above.

### Gene expression assays

*Mo* growth in the leaves of IRBLb-B was quantified on the basis of the ratio between the copy number of a *Mo* sequence (*Pot2*) and the rice *Ubi* gene (GenBank accession D12629), estimated using qPCR^54^. DNA samples were obtained from infected tissue five days post inoculation. The measurement of *AvrPib* transcript abundance was based on total RNA sampled from IRBLb-B leaves inoculated by isolates harboring either *AvrPib*^T38G^ (Mab3–3), *AvrPib*^D1-66^ (Mab12–8) or *AvrPib*^WT^ (b3) at either six, 12, 24 and 72 hpi. The quality of the RNA was assessed^7^, and cDNA was synthesized using M-MLV reverse transcriptase (Promega, Madison, WI, USA) following the manufacturer’s protocol. The *Mo Actin* gene (GenBank accession MGG_03982) was used as the reference sequence, and the *AvrPib* allele-specific primer pair RT-AvrPib-F/R (Supplementary Table S1) employed to amplify *AvrPib* transcripts. Relative transcription levels were analyzed using the 2^–ΔΔCt^ method^55^, setting the abundance of transcript in the *AvrPib*^WT^ isolate at 6 hpi to 1. The data points in both experiments were based on two biological replicates, each subjected to three technical replicates.

## Additional Information

**How to cite this article**: Zhang, S. *et al.* Function and evolution of *Magnaporthe oryzae* avirulence gene *AvrPib* responding to the rice blast resistance gene *Pib. Sci. Rep.* **5**, 11642; doi: 10.1038/srep11642 (2015).

## Supplementary Material

Supplementary Information
