# Supplementary material for: Function and evolution of Magnaporthe oryzae avirulence gene AvrPib responding to the rice blast resistance gene Pib
Source: Sci Rep. 2015 Jun 25;5:11642. doi: 10.1038/srep11642 (PMC5387869; doi:10.1038/srep11642)
Supplement: Supplementary Information [file srep11642-s1.pdf]

# **Molecular mechanisms underlying the function of *Magnaporthe oryzae AvrPib* as revealed by population genetic, comparative genomic and mutagenic approaches**

**Shulin Zhang, Ling Wang, Weihuai Wu, Liyun He, Xianfeng Yang, and Qinghua Pan**

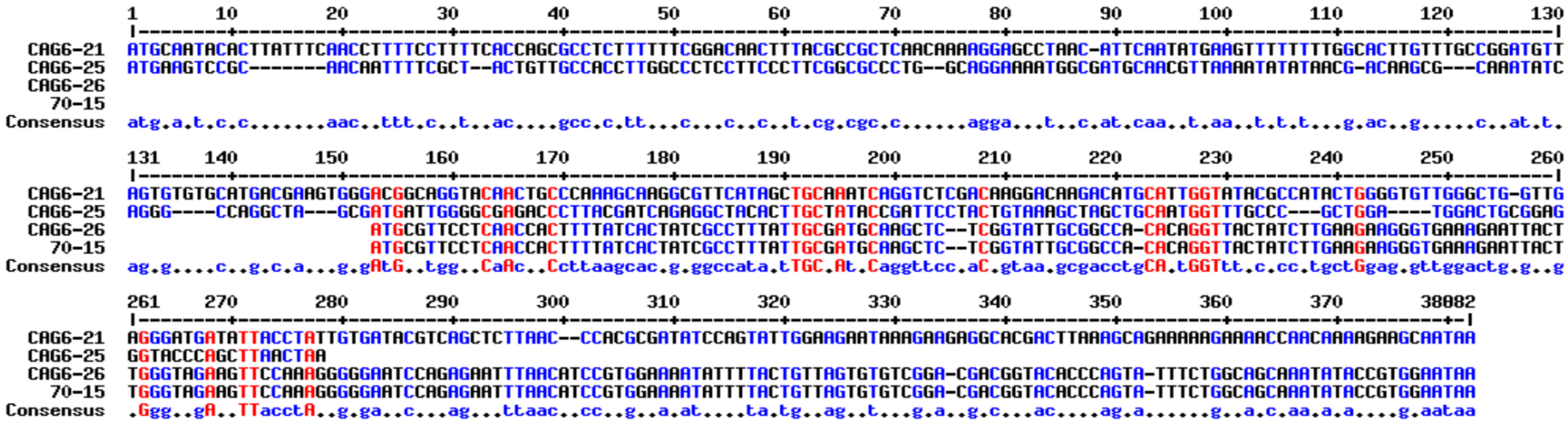

**Figure S1** Alignment of the CDSs of three candidate *AvrPib* genes.

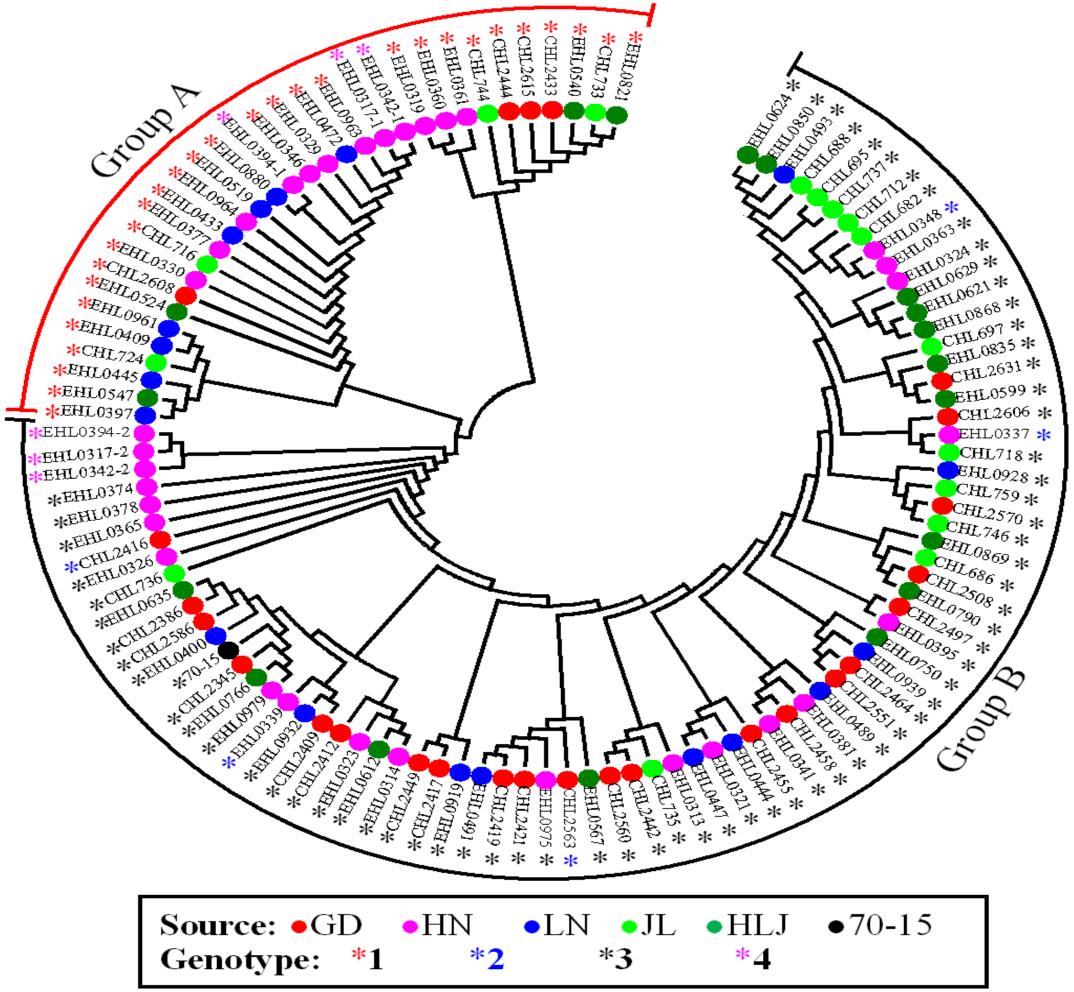


**Figure S2** MEGA6-based phylogenetic analysis of 108 *Mo* isolates, based on the sequences of the gene fragment between positions -710 and +530. The two fragments harbored by isolates of genotype 4 are numbered with the suffix -1 and -2.

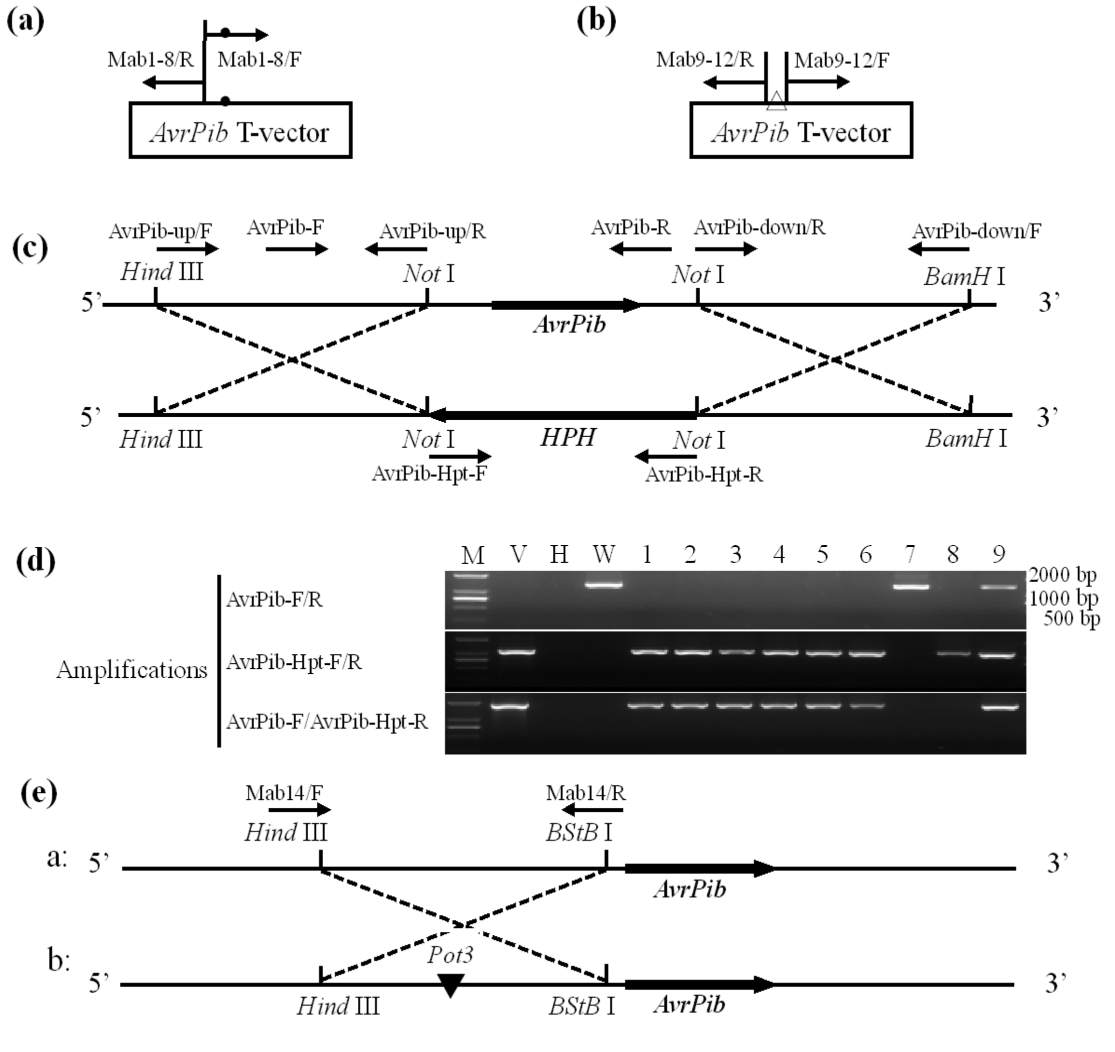


**Figure S3** The reconstruction of *AvrPib* alleles. (a) Targeted nucleotide variant(s) incorporated in the forward primer, with the T-plasmid harboring *AvrPib* used as the template for generating alleles Mab1 through Mab8. (b) Site-directed deletion used to generate alleles Mab9 through Mab12; the targeted nucleotides were omitted from each primer, with the T-plasmid harboring *AvrPib* used as the template. (c) *AvrPib*-directed replacement mutation used to generate the Mab13 allele, in which *AvrPib* was replaced by an *Hpt* cassette. (d) Triple PCR-based confirmation of gene replacement mutants. Amplification based on the three primer pairs AvrPib-F/R, AvrPib-Hpt-F/R, and AvrPib-F/AvrPib-Hpt-R (as indicated in (c)) showed that #1 through #6 were genuine mutants, while #7 through #9 were incorrect. (e) Domain swapping based on the *Pot3* transposon used to generate the Mab14 allele, in which the *Pot3* sequence in CHL724 was introduced into the 5' sequence of CHL42. Appropriate restriction enzyme recognition sites indicated. M: size marker, V: vector (positive control), H: $H_2O$ (negative control), W: isolate CHL42.

**Table S1 PCR primer sequences used in the study**

| Primer name | Forward primer | Enzyme site | Reverse primer | Enzyme site | Use of primers |
|---|---|---|---|---|---|
| MS6-24 | GCGAGTTTACAGGTTTAGCA | | TTTGGGGCTAATAACAGACC | | **Gene mapping** |
| MS6-3 | CCACATCGCAGCCTCACTTG | | CCAGTCGGTTCCAGGTCTTG | | |
| MS6-17 | CAAGAATGCAGAGAACCACAG | | GTTTTCCGCTGTGTAAATAGA | | |
| MS6-7 | CCAAGGCACCAGCACAGACC | | CAGAAGATGCCTCGGAGATC | | |
| MS6-10 | CAACATGTGGTGTCTGATCC | | GCAGGGCCCACTACACATGT | | |
| CAG6-23 | TGCGTTGTGGCGGTAGTCA | | GTTTGTCTGGTCAGCGTCAC | | |
| MS3-5 | GAGTTTGAGACCTTGCGATT | | GGCGGCAAGTCGCTGAAGG | | |
| MS6-34 | CGGATGATGGATGAATTGGC | | AGTGCCGATGCTCCAGAAT | | |
| CAG6-21 | ACCTCGCATTGTCGGCATTC | | TACACCGAGGAACTTGCTTGG | | |
| CAG6-26 | GGACAAGGGAGGCAAATCTAAC | | ATGCCGACAATGCGAGGTAT | | |
| CAG6-25 | ATGCGATTTCTGCGTTGCTA | | CCGAGATACAAAACGCTACCCT | | |
| MS3-26 | GAAGGCGACAAGTGGCAGT | | CTCGTCTGGTATCAGTTGCC | | |
| MS3-6.16 | CGAATATGCAAATGTAAGTT | | GAGTACTTGCCAGAGAGAA | | |
| MS3-6.15 | ATTTCGGTTGATCGTGTTGG | | GCCACCTGTTTCTCCAA | | |
| MS3-6.13 | CCCAAGGTAGGTACGAGAAA | | TTTCTACTGCCAGACAAG | | |
| | | | | | **Gene cloning** |
| b1 | TTGGCGCGCCCGTTTGGTTACGTTCACCCTTG | *Asc*I | TAGGCGCGCCTCGACTACATCCCCATGACGC | *Asc*I | |
| b2 | TTGGCGCGCCGCCATCAACGAGACCACAGCCCAC | *Asc*I | TTGGCGCGCCACTGCGGGAATGCCGACAATGCGAG | *Asc*I | |
| b3 | ATGGCGCGCCGGAGAATGAGCTGCGAGGTC | *Asc*I | TAGGCGCGCCACTGCGGGAATGCCGACAATGCGAG | *Asc*I | |
| | | | | | **Genotyping** |
| AvrPibF1/R1 | GGACAAGGGAGGCAAATCTAAC | | ATGCCGACAATGCGAGGTAT | | |
| AvrPibF2/R2 | TGGAGAAGACTTTGATGC | | GAACGCATAATGGCAACTA | | |
| | | | | | **Mutations** |
| Mab-1 | T**T**TTGCGATGCAAGCTCTCGGTAT | | AAGGCGATAGTGATAAAAGTGG | | |
| Mab-2 | T**C**TTGCGATGCAAGCTCTCGGTAT | | AAGGCGATAGTGATAAAAGTGG | | |
| Mab-3 | TA**G**TGCGATGCAAGCTCTCGGTAT | | AAGGCGATAGTGATAAAAGTGG | | |
| Mab-4 | TTTATCACTATCGCCTTTA**C**TGC | | AGTGGTTGAGGAACGCATAAT | | |
| Mab-5 | CTTT**TG**TGCGATGCAAGCTCT | | GCGATAGTGATAAAAGTGGTT | | |

| | | | | | |
|---|---|---|---|---|---|
| Mab-6 | TTTTATCACTATCGCCTTTT**TC**TGC | | GTGGTTGAGGAACGCATAAT | | |
| Mab-7 | CTTT**CG**TGCGATGCAAGCTCT | | GCGATAGTGATAAAAGTGGTT | | |
| Mab-8 | CTTT**CC**TGCGATGCAAGCTCT | | GCGATAGTGATAAAAGTGGTT | | |
| Mab-9 | TTTTATCACTATCGCCTTTT**GC** | | GTGGTTGAGGAACGCATAAT | | |
| Mab-10 | ATTGCGATGCAAGCTCTCGGTATT | | AATGGCAACTAATTTAATTGTATA | | |
| Mab-11 | ACACAGGTTACTATCTTGAA | | AATAAAGGCGATAGTGATAAAAGTGG | | |
| Mab-12 | ACACAGGTTACTATCTTGAAGAAGGGT | | AATGGCAACTAATTTAATTGTATA | | |
| Mab15 | TATTTTACT**A**TTAGTGTGTCGGAC | | TTTTCCACGGATGTTAAATTCTCT | | |
| Mab16 | GAATCCAGA**A**AATTTAACATCCGTG | | CCCCTTTGGAACTTCTACC | | |
| | | | | | **Gene knock out (Mab13)** |
| AvrPib-up | GCAGCGGCCGCAGACTCAAAGTTCCATAGC | *Not*I | CATAAGCTT AAACAAGGGATGCTGACTAC | *Hind*III | |
| AvrPib-down | GCAGGATCCGTTAGTACCTACAGCTCACAAG | *BamH*I | GCAGCGGCCGCGCTGGATGGTTATTGCGAT | *Not*I | |
| AvrPib-Hpt | GCAGCGGCCGCTATTGAAGGAGCATTTTTGG | *Not*I | GTAGCGGCCGCGCTCTTGTTCGGTCGGCATC | *Not*I | |
| AvrPib | GGACAAGGGAGGCAAATCTAAC | | ATACCTCGCATTGTCGGCAT | | |
| | | | | | **Pot3 domain-swapping** |
| Mab14 | TT AAGCTT AGACTGGCGTCGTGACTGGA | *Hind* III | GAACGCATAATGGCAACTA | *BstB* I | |
| | | | | | **qPCR analysis** |
| Pot2 | ACGACCCGTCTTTACTTATTTGG | | AAGTACGCTTGGTTTTGTTGGAT | | |
| ubiquitin | AACCAGCTGAGGCCCAAGA | | ACGATTGATTTAACCAGTCCATGA | | |
| | | | | | **RT-PCR analysis** |
| Actin | GCGGTTACACCTCTCTACCAC | | AGTCTGGATCTCCTGCTCAAAG | | |
| RT-AvrPib | GGGTAGAAGTTCCAAAGGGG | | TCCACGGTATATTTGCTGCCA | | |

Note: Sequences underlined indicate restrict sites for plasmid constructions, and those boldfaced indicate mutation sites.

**Table S2** Details of the 300 *Mo* isolates collected from five provinces of China, and the 12 chosen isolates, which are pathogenic on grass species other than rice

| Isolate | Growing area [a] | Sampled cultivar | Sampled county | Sampled time[b] | *Pib*-infection type | Genotype [c] | | | Mutation in CDS [d] | *AvrPib* resequencing [e] |
|---|---|---|---|---|---|---|---|---|---|---|
| | | | | | | *AvrPib*F1/R1 | *AvrPib*F2/R2 | Integrated | | |
| Guangdong (GD) population (V/3: 13) | | | | | | | | | | 26 |
| CHL2345 | GD I | Mabayinzhan | Qujiang | 2008E | A | 3 | 3 | 3 | | ✓ |
| CHL2357 | GD I | Shanyou 428 | Wengyuan | 2008E | V | 1 | 1 | 1 | | |
| CHL2370 | GD II | Jinshanyou 3182 | Longchuan | 2008E | V | 1 | 0 | 1 | | |
| CHL2375 | GD I | Shanyou 253 | Renhua | 2008E | V | 1 | 1 | 1 | | |
| CHL2384 | GD I | Changguidao | Lianping | 2008E | V | 0 | 1 | 1 | | |
| CHL2386 | GD II | Fengsimiao | Gaoyao | 2008E | V | 3 | 3 | 3 | $D^{37\text{-}38}$, A137G (E46G) | ✓ |
| CHL2387 | GD II | Shanyou 428 | Meixian | 2008E | V | 0 | 1 | 1 | | |
| CHL2394 | GD II | Nuo | Meixian | 2008E | V | 0 | 0 | 0 | | |
| CHL2401 | GD II | Huayougui 99 | Wuhua | 2008E | V | 1 | 0 | 1 | | |
| CHL2409 | GD II | Zhongerruanzhan | Longmen | 2008E | V | 3 | 3 | 3 | $D^{37\text{-}38}$ | ✓ |
| CHL2412 | GD II | Shanyougui 99 | Fengkai | 2008E | V | 3 | 3 | 3 | $D^{37\text{-}38}$ | ✓ |
| CHL2416 | GD II | Meixiangzhan | Xinxing | 2008E | V | 2 | 2 | 2 | | ✓ |
| CHL2417 | GD II | Tianyou 290 | Xingning | 2008E | MV | 0 | 3 | 3 | T38G (I13S)，G166A (V56I) | ✓ |
| CHL2419 | GD II | Changguidao | Jiexi | 2008E | V | 3 | 3 | 3 | $D^{37\text{-}38}$ | ✓ |
| CHL2421 | GD III | Sanyinian | Yangjiang | 2008E | V | 3 | 3 | 3 | $D^{37\text{-}38}$ | ✓ |
| CHL2426 | GD III | Youyou 389 | Xinyi | 2008E | V | 0 | 1 | 1 | | |
| CHL2433 | GD III | Huayou 86 | Xinyi | 2008E | V | 1 | 1 | 1 | | ✓ |
| CHL2436 | GD III | Huayou 86 | Xinyi | 2008E | V | 0 | 1 | 1 | | |
| CHL2438 | GD III | Changguidao | Gaozhou | 2008E | V | 0 | 0 | 0 | | |
| CHL2441 | GD III | Zajiaodao | Gaozhou | 2008E | V | 1 | 1 | 1 | | |
| CHL2442 | GD III | Tianyou 998 | Haifeng | 2008E | A | 3 | 3 | 3 | | ✓ |
| CHL2444 | GD III | Guangyinzhan | Foshan | 2008E | V | 1 | 0 | 1 | | ✓ |
| CHL2446 | GD III | Shuanghuangzhan | Jiedong | 2008E | V | 1 | 1 | 1 | | |

| | | | | | | | | | | |
|---|---|---|---|---|---|---|---|---|---|---|
| CHL2448 | GD III | Teyou 721 | Chaoan | 2008E | V | 1 | 1 | 1 | | |
| CHL2449 | GD II | Zengyouzhan | Zengcheng | 2008E | V | 3 | 3 | 3 | D[37-38] | ✓ |
| CHL2452 | GD III | Fengqin | Lianjiang | 2008E | V | 1 | 1 | 1 | | |
| CHL2455 | GD III | Bairizao | Huidong | 2008E | V | 3 | 3 | 3 | D[37-38] | ✓ |
| CHL2457 | GD III | Zhanyou 226 | Huidong | 2008E | V | 1 | 1 | 1 | | |
| CHL2458 | GD III | Teyou 721 | Yangchun | 2008E | V | 3 | 3 | 3 | D[37-38] | ✓ |
| CHL2464 | GD III | Taishanbai | Kaiping | 2008E | A | 3 | 3 | 3 | | ✓ |
| CHL2467 | GD I | Meixiangzhan | Renhua | 2008L | V | 2 | 2 | 2 | | |
| CHL2486 | GD I | Fengyou 998 | Lechang | 2008L | V | 1 | 1 | 1 | | |
| CHL2490 | GD I | Guodao No.1 | Ruyuan | 2008L | V | 1 | 1 | 1 | | |
| CHL2492 | GD I | Yueyou 360 | Nanxiong | 2008L | V | 0 | 0 | 0 | | |
| CHL2497 | GD I | Meixiangzhan | Qujiang | 2008L | A | 3 | 3 | 3 | | ✓ |
| CHL2508 | GD I | Youyou 308 | Wengyuan | 2008L | V | 3 | 3 | 3 | D[37-38] | ✓ |
| CHL2509 | GD I | Shanyou 89 | Pingyuan | 2008L | V | 1 | 1 | 1 | | |
| CHL2513 | GD I | Shanyou 46 | Xinfeng | 2008L | V | 0 | 1 | 1 | | |
| CHL2516 | GD I | Shanyou 82 | Jiaoling | 2008L | V | 1 | 1 | 1 | | |
| CHL2518 | GD II | Yuxiang 88 | Longchuan | 2008L | V | 0 | 1 | 1 | | |
| CHL2534 | GD II | Shanyou 122 | Meixian | 2008L | V | 1 | 1 | 1 | | |
| CHL2547 | GD II | Tianyou 368 | Fengshun | 2008L | V | 1 | 1 | 1 | | |
| CHL2549 | GD II | Shanyou 122 | Dapu | 2008L | V | 0 | 1 | 1 | | |
| CHL2551 | GD II | Boyouyun 3 | Zijin | 2008L | V | 3 | 3 | 3 | D[37-38] | ✓ |
| CHL2560 | GD III | Huanghuazhan | Boluo | 2008L | A | 3 | 3 | 3 | | ✓ |
| CHL2561 | GD II | Longyou 665 | Fougang | 2008L | V | 0 | 1 | 1 | | |
| CHL2563 | GD III | Zhanzinuo | Huidong | 2008L | V | 2 | 2 | 2 | | ✓ |
| CHL2570 | GD II | Yesizhan | Longmen | 2008L | V | 3 | 3 | 3 | D[37-38] | ✓ |
| CHL2581 | GD II | Qiuyou 452 | Qingxin | 2008L | V | 1 | 0 | 1 | | |
| CHL2584 | GD II | Zayounuo | Huaiji | 2008L | V | 1 | 1 | 1 | | |
| CHL2586 | GD II | Zhenguizhong | Yingde | 2008L | A | 3 | 3 | 3 | | ✓ |

| | | | | | | | | | | |
|---|---|---|---|---|---|---|---|---|---|---|
| CHL2593 | GD II | Qiuyou 3008 | Yunfu | 2008L | V | 1 | 0 | 1 | | |
| CHL2600 | GD II | Qiuyou 3008 | Deqing | 2008L | V | 1 | 0 | 1 | | |
| CHL2604 | GD III | Guinongzhan | Haifeng | 2008L | V | 1 | 1 | 1 | | |
| CHL2606 | GD III | Xiaonongzhan | Zhuhai | 2008L | A | 3 | 3 | 3 | | ✓ |
| CHL2608 | GD III | Taiwanyouzhan | Yangchun | 2008L | V | 1 | 3 | 1 | | ✓ |
| CHL2615 | GD III | Bo II You 15 | Lianjiang | 2008L | V | 1 | 1 | 1 | | ✓ |
| CHL2626 | GD III | Longyou 673 | Xinyi | 2008L | V | 1 | 1 | 1 | | |
| CHL2631 | GD III | Shanyou 3550 | Gaozhou | 2008L | V | 3 | 3 | 3 | $D^{37-38}$, T201C (S67S) | ✓ |
| CHL2636 | GD IV | Bo II You 15 | Suixi | 2008L | V | 1 | 1 | 1 | | |
| Hunan (HN) population (V/3: 8) | | | | | | | | | | 30 |
| EHL0312 | HN I | Tefu No.7 | Taoyuan | 2007 | V | 1 | 1 | 1 | | |
| EHL0313 | HN I | Tefu No.7 | Taoyuan | 2007 | A | 3 | 3 | 3 | | ✓ |
| EHL0314 | HN II | Weiyou 402 | Anren | 2007 | V | 3 | 3 | 3 | $D^{37-38}$ | ✓ |
| EHL0317 | HN II | Weiyou 402 | Anren | 2007 | V | 4 | 4 | 4 | $D^{39-66}$, A120G (P40P) | ✓ |
| EHL0319 | HN II | Liangyou 287 | Liling | 2007 | V | 1 | 3 | 1 | | ✓ |
| EHL0321 | HN II | V402 | Hengnan | 2007 | V | 3 | 3 | 3 | $D^{37-38}$ | ✓ |
| EHL0323 | HN II | V233 | Hengdong | 2007 | V | 3 | 3 | 3 | $D^{37-38}$ | ✓ |
| EHL0324 | HN II | I You 402 | Hengdong | 2007 | V | 3 | 3 | 3 | $D^{37-38}$ | ✓ |
| EHL0326 | HN II | I You 402 | Hengnan | 2007 | V | 3 | 3 | 3 | $D^{37-38}$, C207T (S69S) | ✓ |
| EHL0327 | HN II | Zhuliangyou 30 | Dongan | 2007 | V | 1 | 1 | 1 | | |
| EHL0329 | HN II | Douyuanyou 299 | Dongan | 2007 | V | 1 | 1 | 1 | G136A (E46K) | ✓ |
| EHL0330 | HN II | T You 99 | Dongan | 2007 | V | 1 | 1 | 1 | | ✓ |
| EHL0331 | HN II | Xianfeng 501 | Dongan | 2007 | V | 1 | 1 | 1 | | |
| EHL0337 | HN II | Jinyou 71 | Dongan | 2007 | V | 2 | 2 | 2 | | ✓ |
| EHL0338 | HN II | Jinyou 898 | Dongan | 2007 | V | 0 | 0 | 0 | | |
| EHL0339 | HN II | Nongping 001 | Dongan | 2007 | V | 2 | 2 | 2 | | ✓ |
| EHL0340 | HN II | Weiyou 507 | Dongan | 2007 | V | 0 | 0 | 0 | | |

| | | | | | | | | | | |
|---|---|---|---|---|---|---|---|---|---|---|
| EHL0341 | HN II | Weiyou 298 | Dongan | 2007 | V | 3 | 3 | 3 | $D^{37-38}$ | ✓ |
| EHL0342 | HN II | Weiyou 463 | Dongan | 2007 | V | 4 | 4 | 4 | $D^{39-66}$, G90C (K30N),A120G(P40P),G123A(K41K) | ✓ |
| EHL0345 | HN II | T You 213 | Dongan | 2007 | V | 1 | 1 | 1 | | |
| EHL0346 | HN II | T You 103 | Dongan | 2007 | V | 1 | 3 | 1 | | ✓ |
| EHL0348 | HN II | Jinyou 103 | Dongan | 2007 | V | 2 | 2 | 2 | | ✓ |
| EHL0349 | HN II | Jinyou 402 | Dongan | 2007 | V | 1 | 1 | 1 | | |
| EHL0350 | HN II | Xianfeng No.1 | Dongan | 2007 | V | 0 | 1 | 1 | | |
| EHL0353 | HN II | Nongfengyou 909 | Dongan | 2007 | V | 1 | 1 | 1 | | |
| EHL0354 | HN II | Jinyou 821 | Dongan | 2007 | V | 0 | 1 | 1 | | |
| EHL0355 | HN II | Weiyou 463 | Dongan | 2007 | V | 1 | 1 | 1 | | |
| EHL0356 | HN II | Zhongyou No.1 | Dongan | 2007 | V | 1 | 1 | 1 | | |
| EHL0360 | HN II | Zhongyou No.1 | Dongan | 2007 | V | 1 | 3 | 1 | | ✓ |
| EHL0361 | HN II | Zhuliangyou 02 | Dongan | 2007 | V | 1 | 3 | 1 | | ✓ |
| EHL0363 | HN II | Xiangnuo | Dongan | 2007 | A | 3 | 3 | 3 | | ✓ |
| EHL0364 | HN II | T You 706 | Dongan | 2007 | V | 0 | 1 | 1 | | |
| EHL0365 | HN II | Zhongyou 493 | Dongan | 2007 | A | 3 | 3 | 3 | | ✓ |
| EHL0367 | HN II | Jinyou 463 | Dongan | 2007 | V | 1 | 1 | 1 | | |
| EHL0368 | HN II | Zhuliangyou 83 | Dongan | 2007 | V | 0 | 1 | 1 | | |
| EHL0369 | HN III | Jinyou 899 | Daoxian | 2007 | V | 1 | 1 | 1 | | |
| EHL0370 | HN III | Jinyou 463 | Daoxian | 2007 | V | 0 | 0 | 0 | | |
| EHL0372 | HN III | Nuogu | Daoxian | 2007 | V | 0 | 0 | 0 | | |
| EHL0373 | HN III | Jinyou 463 | Daoxian | 2007 | V | 1 | 1 | 1 | | |
| EHL0374 | HN III | Shennongdao 101 | Daoxian | 2007 | A | 3 | 3 | 3 | | ✓ |
| EHL0377 | HN I | Jing No.5 | Yiyang | 2007 | V | 1 | 3 | 1 | | ✓ |
| EHL0378 | HN I | Wanxiang No. 13 | Taojiang | 2007 | A | 3 | 3 | 3 | | ✓ |
| EHL0379 | HN I | Pin No. 3 | Taojiang | 2007 | V | 0 | 1 | 1 | | |
| EHL0380 | HN I | Wannuo | Yiyang | 2007 | V | 1 | 1 | 1 | | |
| EHL0381 | HN I | Wannuo | Yiyang | 2007 | A | 3 | 3 | 3 | | ✓ |

| | | | | | | | | | | |
|---|---|---|---|---|---|---|---|---|---|---|
| EHL0382 | HN I | Nuogu | YueYang | 2007 | V | 0 | 0 | 0 | | |
| EHL0385 | HN II | II You 58 | Liuyang | 2007 | V | 0 | 1 | 1 | | |
| EHL0386 | HN II | Minghui 63 | Liuyang | 2007 | V | 1 | 1 | 1 | | |
| EHL0387 | HN II | II You 838 | Liuyang | 2007 | V | 1 | 1 | 1 | | |
| EHL0393 | HN II | Y NO. 232 | Youxian | 2007 | V | 1 | 1 | 1 | | |
| EHL0394 | HN II | Xinshanyou 64 | Anren | 2007 | V | 4 | 4 | 4 | $D^{39-66}$, A120G (P40P) | ✓ |
| EHL0395 | HN IV | Zhongyou 288 | Huaihua | 2007 | A | 3 | 3 | 3 | | ✓ |
| EHL0963 | HN I | Xianghuayou No. 7 | Taojiang | 2008 | V | 1 | 3 | 1 | | ✓ |
| EHL0964 | HN I | Xianghuayou No. 8 | Taojiang | 2008 | V | 1 | 3 | 1 | | ✓ |
| EHL0975 | HN I | Fuyou II | Taojiang | 2008 | V | 3 | 3 | 3 | $D^{37-38}$ | ✓ |
| EHL0979 | HN II | Mixiang No.1 | Changsha | 2008 | V | 3 | 3 | 3 | $D^{37-38}$ | ✓ |
| EHL0982 | HN II | Xiangwanxian No.17 | Changsha | 2008 | V | 0 | 0 | 0 | | |
| EHL0988 | HN II | Chaojidao 527 | Zhuzhou | 2008 | V | 1 | 1 | 1 | | |
| EHL0989 | HN II | Xiangwanxian No.13 | Zhuzhou | 2008 | V | 1 | 1 | 1 | | |
| EHL0990 | HN II | Xiangzaoxian No.24 | Liling | 2008 | V | 0 | 1 | 1 | | |
| Liaoning (LN) population (V/3: 0) | | | | | | | | | | 18 |
| EHL0396 | LN I | Minxi 12 | Zhuanghe | 2006 | A | 3 | 3 | 3 | | |
| EHL0397 | LN I | Chenhong 59 | Zhuanghe | 2006 | V | 1 | 3 | 1 | | ✓ |
| EHL0400 | LN I | Lige | Zhuanghe | 2006 | A | 3 | 3 | 3 | | ✓ |
| EHL0409 | LN I | Yanjing 167 | Donggang | 2006 | V | 1 | 1 | 1 | | ✓ |
| EHL0412 | LN I | Huanghai No.6 | Donggang | 2006 | V | 1 | 1 | 1 | | |
| EHL0431 | LN I | Liaodan 203 | Donggang | 2006 | V | 1 | 1 | 1 | | |
| EHL0433 | LN I | Liaohan 109 | Donggang | 2006 | V | 1 | 1 | 1 | | ✓ |
| EHL0444 | LN II | Chenhong 59 | Pulandian | 2006 | A | 3 | 3 | 3 | | ✓ |
| EHL0445 | LN II | Minxi 12 | Pulandian | 2006 | V | 1 | 1 | 1 | | ✓ |
| EHL0446 | LN II | Gangyu 129 | Pulandian | 2006 | V | 0 | 1 | 1 | | |
| EHL0447 | LN II | Koshihikari | Pulandian | 2006 | A | 3 | 3 | 3 | | ✓ |
| EHL0468 | LN II | Maoyang No.3 | Fengchen | 2006 | V | 1 | 1 | 1 | | |

| | | | | | | | | | |
|---|---|---|---|---|---|---|---|---|---|
| EHL0471 | LN II | Qiao 201-2 | Fengchen | 2006 | V | 1 | 1 | 1 | |
| EHL0472 | LN II | Panjin 78-2 | Fengchen | 2006 | V | 1 | 3 | 1 | ✓ |
| EHL0474 | LN II | Chenhe No.1 | Fengchen | 2006 | V | 1 | 1 | 1 | |
| EHL0484 | LN II | Yuanfeng No.9 | Fushun | 2006 | V | 1 | 1 | 1 | |
| EHL0486 | LN II | Fuyou 504 | Fushun | 2006 | V | 1 | 1 | 1 | |
| EHL0489 | LN II | Shennong 0705 | Fushun | 2006 | A | 3 | 3 | 3 | ✓ |
| EHL0491 | LN II | Jijing 88 | Fushun | 2006 | A | 3 | 3 | 3 | ✓ |
| EHL0492 | LN II | Xin No.2 | Fushun | 2006 | V | 1 | 1 | 1 | |
| EHL0493 | LN II | Shennong 315 | Fushun | 2006 | A | 3 | 3 | 3 | ✓ |
| EHL0496 | LN II | Su 01-18 | Dashiqiao | 2006 | V | 1 | 1 | 1 | |
| EHL0498 | LN II | Yanfeng | Dashiqiao | 2006 | V | 1 | 1 | 1 | |
| EHL0499 | LN II | Yanfeng 47-12 | Dashiqiao | 2006 | V | 1 | 1 | 1 | |
| EHL0500 | LN III | Chenhe No.1 | Panjin | 2006 | V | 1 | 1 | 1 | |
| EHL0503 | LN III | Maoyang No.3 | Panjin | 2006 | V | 1 | 3 | 1 | |
| EHL0505 | LN III | Liaohe 12 | Panjin | 2006 | V | 1 | 1 | 1 | |
| EHL0509 | LN III | Yanjing 228 | Panjin | 2006 | V | 0 | 1 | 1 | |
| EHL0515 | LN III | Liaohe No.1 | Panjin | 2006 | V | 1 | 1 | 1 | |
| EHL0517 | LN III | Huadan 995 | Shenyang | 2006 | V | 1 | 1 | 1 | |
| EHL0519 | LN III | Huadan 995 | Tieling | 2006 | V | 1 | 1 | 1 | ✓ |
| EHL0879 | LN I | Liaoxing No.1 | Dandong | 2007 | V | 0 | 0 | 0 | |
| EHL0880 | LN I | Gangyu No.2 | Dandong | 2007 | V | 1 | 1 | 1 | ✓ |
| EHL0882 | LN I | Chenhe No.1 | Dandong | 2007 | V | 0 | 0 | 0 | |
| EHL0883 | LN I | Maoyang No.5 | Dandong | 2007 | V | 1 | 1 | 1 | |
| EHL0885 | LN I | Liaojing No.9 | Dandong | 2007 | V | 1 | 1 | 1 | |
| EHL0916 | LN II | Heixiangdao | Dalian | 2007 | V | 1 | 1 | 1 | |
| EHL0917 | LN II | Zhuangyan No.8 | Dalian | 2007 | V | 1 | 1 | 1 | |
| EHL0918 | LN II | Koshihikari | Dalian | 2007 | V | 1 | 1 | 1 | |
| EHL0919 | LN II | Yanfeng 47 | Dalian | 2007 | A | 3 | 3 | 3 | ✓ |

| | | | | | | | | | | |
|---|---|---|---|---|---|---|---|---|---|---|
| EHL0921 | LN II | Gangyuan No.8 | Dalian | 2007 | V | 1 | 1 | 1 | | |
| EHL0922 | LN II | Liaonong 06-7 | Qinyuan | 2007 | V | 1 | 1 | 1 | | |
| EHL0924 | LN II | Fu 9906 | Qinyuan | 2007 | V | 0 | 0 | 0 | | |
| EHL0925 | LN II | Fuxing 90 | Qinyuan | 2007 | V | 1 | 1 | 1 | | |
| EHL0926 | LN II | Jijing 88 | Qinyuan | 2007 | V | 0 | 1 | 1 | | |
| EHL0927 | LN II | Daohuaxiang | Qinyuan | 2007 | A | 3 | 3 | 3 | | |
| EHL0928 | LN II | Liaonong 06-7 | Xinbin | 2007 | A | 3 | 3 | 3 | | ✓ |
| EHL0931 | LN II | Jiudao 46 | Xinbin | 2007 | A | 3 | 3 | 3 | | |
| EHL0932 | LN II | Xinyu No.3 | Xinbin | 2007 | A | 3 | 3 | 3 | | ✓ |
| EHL0935 | LN II | Jijing 88 | Xinbin | 2007 | A | 3 | 3 | 3 | | |
| EHL0936 | LN II | Shendao 37 | Xifeng | 2007 | V | 1 | 1 | 1 | | |
| EHL0937 | LN II | Shennong 315 | Xifeng | 2007 | V | 1 | 1 | 1 | | |
| EHL0939 | LN II | Xinyi No.3 | Xifeng | 2007 | A | 3 | 0 | 3 | | ✓ |
| EHL0940 | LN II | Fuxing 90 | Xifeng | 2007 | V | 1 | 1 | 1 | | |
| EHL0941 | LN II | Xinyi No.3 | Kaiyuan | 2007 | V | 1 | 1 | 1 | | |
| EHL0942 | LN II | Shennong 9903 | Kaiyuan | 2007 | V | 0 | 1 | 1 | | |
| EHL0943 | LN II | Huadan No.995 | Kaiyuan | 2007 | V | 1 | 1 | 1 | | |
| EHL0944 | LN II | Fuxing 90 | Kaiyuan | 2007 | V | 1 | 1 | 1 | | |
| EHL0961 | LN IV | Chenhe No.1 | Suizhong | 2007 | V | 1 | 1 | 1 | T173C (V58A) | ✓ |
| EHL0962 | LN IV | Yanjing 218 | Suizhong | 2007 | V | 1 | 1 | 1 | | |
| Jilin (JL) population (V/3: 0) | | | | | | | | | | 16 |
| CHL678 | JL I | Tong 31 | Yongji | 2003 | A | 3 | 3 | 3 | | |
| CHL680 | JL I | Ji 04 | Yongji | 2003 | A | 3 | 3 | 3 | | |
| CHL682 | JL I | Tong 313 | Yongji | 2003 | A | 3 | 3 | 3 | | ✓ |
| CHL684 | JL I | Fengyou 201 | Yongji | 2003 | A | 3 | 3 | 3 | | |
| CHL686 | JL I | Pingjing 1 | Yongji | 2003 | A | 3 | 3 | 3 | | ✓ |
| CHL688 | JL I | Jiu 01 C 6 | Yongji | 2003 | A | 3 | 3 | 3 | | ✓ |
| CHL690 | JL I | Changbai 9 | Shulan | 2003 | A | 3 | 3 | 3 | | |

| | | | | | | | | | |
|---|---|---|---|---|---|---|---|---|---|
| CHL692 | JL I | Dongnong V 7 | Shulan | 2003 | A | 3 | 3 | 3 | |
| CHL693 | JL I | Yan 312 | Shulan | 2003 | A | 3 | 3 | 3 | |
| CHL695 | JL I | Songjing 6 | Shulan | 2003 | A | 3 | 3 | 3 | ✓ |
| CHL697 | JL I | 301 | Shulan | 2003 | A | 3 | 3 | 3 | ✓ |
| CHL699 | JL I | Tongyu 12 | Shulan | 2003 | A | 3 | 3 | 3 | |
| CHL700 | JL I | Wuyou 1 | Shulan | 2003 | A | 3 | 3 | 3 | |
| CHL701 | JL I | 99-35 | Panshi | 2003 | A | 3 | 3 | 3 | |
| CHL702 | JL I | Tongfeng No. 3 | Panshi | 2003 | A | 3 | 3 | 3 | |
| CHL703 | JL I | Ji 01-3628 | Panshi | 2003 | A | 3 | 3 | 3 | |
| CHL704 | JL I | Pingjing No. 2 | Panshi | 2003 | A | 3 | 3 | 3 | |
| CHL705 | JL I | Chang 6 | Panshi | 2003 | A | 3 | 3 | 3 | |
| CHL708 | JL IV | Tongjing 612 | Jiaohe | 2003 | A | 3 | 3 | 3 | |
| CHL710 | JL IV | Yan 313 | Jiaohe | 2003 | A | 3 | 3 | 3 | |
| CHL712 | JL IV | Changbai 9 | Jiaohe | 2003 | A | 3 | 3 | 3 | ✓ |
| CHL714 | JL IV | Wuyou C | Jiaohe | 2003 | A | 3 | 3 | 3 | |
| CHL715 | JL IV | Yannian No. 1 | Jiaohe | 2003 | V | 0 | 1 | 1 | |
| CHL716 | JL IV | Jiudao 16 | Jiaohe | 2003 | V | 1 | 1 | 1 | ✓ |
| CHL718 | JL IV | Changlixiang 12 | Jiaohe | 2003 | A | 3 | 3 | 3 | ✓ |
| CHL721 | JL I | Jiu 01 D 13 | Jilin | 2003 | A | 3 | 3 | 3 | |
| CHL722 | JL I | Ji T620-4 | Jilin | 2003 | A | 3 | 3 | 3 | |
| CHL724 | JL I | T 02 | Jilin | 2003 | V | 1 | 1 | 1 | ✓ |
| CHL725 | JL I | Tongjing 790 B | Jilin | 2003 | V | 1 | 1 | 1 | |
| CHL730 | JL I | Tongnian 3 | Jilin | 2003 | A | 3 | 3 | 3 | |
| CHL731 | JL I | Tongyu 308 | Jilin | 2003 | A | 3 | 3 | 3 | |
| CHL733 | JL I | Tongnian 23 | Meihekou | 2003 | V | 1 | 1 | 1 | ✓ |
| CHL734 | JL I | Tong 3158 | Liuhe | 2003 | A | 3 | 3 | 3 | |
| CHL735 | JL I | Daliwang | Liuhe | 2003 | A | 3 | 3 | 3 | ✓ |
| CHL736 | JL I | Unknown | Lishu | 2003 | A | 3 | 3 | 3 | ✓ |

| | | | | | | | | | |
|---|---|---|---|---|---|---|---|---|---|
| CHL737 | JL I | Unknown | Yitong | 2003 | A | 3 | 3 | 3 | ✓ |
| CHL739 | JL I | 182 | Jiutai | 2003 | V | 0 | 1 | 1 | |
| CHL740 | JL I | 183 | Jiutai | 2003 | V | 0 | 1 | 1 | |
| CHL741 | JL I | No. 55 | Jiutai | 2003 | V | 1 | 1 | 1 | |
| CHL742 | JL I | No. 55 | Jiutai | 2003 | V | 1 | 1 | 1 | |
| CHL743 | JL I | No. 52 | Jiutai | 2003 | V | 1 | 1 | 1 | |
| CHL744 | JL I | Tong 793 | Dehui | 2003 | V | 1 | 1 | 1 | ✓ |
| CHL745 | JL I | Tong 190 | Dehui | 2003 | V | 1 | 1 | 1 | |
| CHL746 | JL I | Menggudao | Dongfeng | 2003 | A | 3 | 3 | 3 | ✓ |
| CHL748 | JL I | Changxiangsi | Dongfeng | 2003 | A | 3 | 3 | 3 | |
| CHL749 | JL I | Tongjing 769 | Dongfeng | 2003 | V | 1 | 1 | 1 | |
| CHL751 | JL I | Tongyin 58 | Dongfeng | 2003 | A | 3 | 3 | 3 | |
| CHL752 | JL I | Zupei 11 | Dongfeng | 2003 | A | 3 | 3 | 3 | |
| CHL754 | JL I | Chaochan 2 | Gongzhuling | 2003 | V | 1 | 1 | 1 | |
| CHL755 | JL I | Unknown | Yitong | 2003 | A | 3 | 3 | 3 | |
| CHL756 | JL I | Xueguang | Yongji | 2002 | A | 3 | 3 | 3 | |
| CHL757 | JL IV | Unknown | Jiaohe | 2002 | A | 3 | 3 | 3 | |
| CHL759 | JL III | Shangyu No. 397 | Helong | 2002 | A | 3 | 3 | 3 | ✓ |
| CHL760 | JL I | Menggudao | Meihekou | 2002 | A | 3 | 3 | 3 | |
| CHL761 | JL I | 403 | Liuhe | 2002 | A | 3 | 3 | 3 | |
| CHL762 | JL I | 112 | Liuhe | 2002 | A | 3 | 3 | 3 | |
| CHL764 | JL I | Tong 458 | Dongfeng | 2002 | A | 3 | 3 | 3 | |
| CHL765 | JL I | Unknown | Gongzhuling | 2002 | A | 3 | 3 | 3 | |
| CHL766 | JL I | Unknown | Gongzhuling | 2002 | A | 3 | 3 | 3 | |
| CHL767 | JL I | 939 | Baichen | 2002 | A | 3 | 3 | 3 | |
| Heilongjiang (HLJ) population (V/3: 0) | | | | | | | | | 18 |
| EHL0524 | HLJ III | Songjing 7 | Zhaoyuan | 2006 | V | 1 | 1 | 1 | ✓ |
| EHL0527 | HLJ II | Kendao 10 | Anqin | 2006 | V | 1 | 1 | 1 | |

| | | | | | | | | | | |
|---|---|---|---|---|---|---|---|---|---|---|
| EHL0532 | HLJ II | Song 98-131 | Anqin | 2006 | V | 1 | 1 | 1 | | |
| EHL0540 | HLJ I | Songjing 10 | Achen | 2006 | V | 1 | 1 | 1 | | ✓ |
| EHL0547 | HLJ II | Kendao 12 | Tonghe | 2006 | V | 1 | 1 | 1 | | ✓ |
| EHL0555 | HLJ II | Kuyuku 131 | Tonghe | 2006 | A | 3 | 3 | 3 | | |
| EHL0557 | HLJ II | Suijing No.4 | Tonghe | 2006 | A | 3 | 3 | 3 | | |
| EHL0559 | HLJ II | Dongnong 220 | Shangzhi | 2006 | A | 3 | 3 | 3 | | |
| EHL0560 | HLJ II | Puyou 18 | Shangzhi | 2006 | V | 1 | 1 | 1 | | |
| EHL0567 | HLJ II | Wuyoudao No.3 | Shangzhi | 2006 | A | 3 | 3 | 3 | | ✓ |
| EHL0568 | HLJ II | Wuyoudao No.3 | Shangzhi | 2006 | A | 3 | 3 | 3 | | |
| EHL0570 | HLJ II | Mudanjiang 19 | Shangzhi | 2006 | A | 3 | 3 | 3 | | |
| EHL0571 | HLJ II | Kendao 10 | Shangzhi | 2006 | A | 3 | 3 | 3 | | |
| EHL0592 | HLJ II | Shangyu 397 | Muling | 2006 | A | 3 | 3 | 3 | | |
| EHL0598 | HLJ III | Punian No.7 | Hulin | 2006 | A | 3 | 3 | 3 | | |
| EHL0599 | HLJ III | Kenjiandao No.6 | Hulin | 2006 | A | 3 | 3 | 3 | | ✓ |
| EHL0603 | HLJ III | Kuyuku 131 | Hulin | 2006 | V | 1 | 1 | 1 | | |
| EHL0611 | HLJ III | Kendao 12 | Hulin | 2006 | A | 3 | 3 | 3 | | |
| EHL0612 | HLJ II | Kuyuku 131 | Mishan | 2006 | A | 3 | 3 | 3 | | ✓ |
| EHL0615 | HLJ II | Suijing No.4 | Mishan | 2006 | A | 3 | 3 | 3 | | |
| EHL0618 | HLJ II | Kuyuku 131 | Mishan | 2006 | A | 3 | 3 | 3 | | |
| EHL0621 | HLJ II | Longdun 02-318 | Jidong | 2006 | A | 3 | 3 | 3 | T160C (F54L) | ✓ |
| EHL0624 | HLJ II | Kuyuku 131 | Tangyuan | 2006 | A | 3 | 3 | 3 | | ✓ |
| EHL0625 | HLJ II | Kuyuku 131 | Huachuan | 2006 | V | 1 | 1 | 1 | | |
| EHL0629 | HLJ II | Longdun 102 | Jiamusi | 2006 | A | 3 | 3 | 3 | | ✓ |
| EHL0632 | HLJ II | Kuyuku 131 | Jiamusi | 2006 | A | 3 | 3 | 3 | | |
| EHL0635 | HLJ II | Kuyuku 131 | Baoqin | 2006 | A | 3 | 3 | 3 | | ✓ |
| EHL0636 | HLJ II | Kuyuku 131 | Jixian | 2006 | A | 3 | 3 | 3 | | |
| EHL0750 | HLJ I | Kuyuku 131 | Mudanjiang | 2006 | A | 3 | 3 | 3 | | ✓ |
| EHL0755 | HLJ II | Kuyuku 131 | Tangyuan | 2006 | A | 3 | 3 | 3 | | |

| | | | | | | | | | | |
|---|---|---|---|---|---|---|---|---|---|---|
| EHL0757 | HLJ II | Kendao No.6 | Tangyuan | 2006 | A | 3 | 3 | 3 | | |
| EHL0764 | HLJ II | Kendao No.11 | Qinan | 2006 | A | 3 | 3 | 3 | | |
| EHL0766 | HLJ II | Kuyuku 131 | Qinan | 2006 | A | 3 | 3 | 3 | | ✓ |
| EHL0770 | HLJ II | Longjing 17 | Qinan | 2006 | A | 3 | 3 | 3 | | |
| EHL0776 | HLJ I | Kendao No.6 | Mudanjiang | 2006 | A | 3 | 3 | 3 | | |
| EHL0779 | HLJ I | Kuyuku 131 | Mudanjiang | 2006 | A | 3 | 3 | 3 | | |
| EHL0781 | HLJ I | Longjing 17 | Mudanjiang | 2006 | A | 3 | 3 | 3 | | |
| EHL0787 | HLJ III | Kenjiandao 13 | Jiansanjiang | 2006 | A | 3 | 3 | 3 | | |
| EHL0790 | HLJ III | Kuyuku 131 | Jiansanjiang | 2006 | A | 3 | 3 | 3 | | ✓ |
| EHL0794 | HLJ I | Jite 639 | Wuchang | 2007 | A | 3 | 3 | 3 | | |
| EHL0798 | HLJ III | Songjing 3 | Zhaodong | 2007 | V | 0 | 1 | 1 | | |
| EHL0801 | HLJ III | Songjing 12 | Zhaodong | 2007 | V | 1 | 1 | 1 | | |
| EHL0814 | HLJ III | Jijing 106 | Zhaodong | 2007 | V | 1 | 1 | 1 | | |
| EHL0821 | HLJ I | Songjing 5 | Shuangchen | 2007 | V | 1 | 1 | 1 | T116C (V39A) | ✓ |
| EHL0828 | HLJ I | Songjing 6 | Shuangchen | 2007 | V | 1 | 1 | 1 | | |
| EHL0830 | HLJ II | Kendao 12 | Fangzheng | 2007 | V | 1 | 1 | 1 | | |
| EHL0834 | HLJ I | Menggudao | Mudanjiang | 2007 | A | 3 | 3 | 3 | | |
| EHL0835 | HLJ I | Menggudao | Mudanjiang | 2007 | A | 3 | 3 | 3 | | ✓ |
| EHL0836 | HLJ I | Shangyu 397 | Mudanjiang | 2007 | A | 3 | 3 | 3 | | |
| EHL0837 | HLJ I | Shangyu 397 | Mudanjiang | 2007 | A | 3 | 3 | 3 | | |
| EHL0849 | HLJ II | Kuyuku 131 | Muling | 2007 | A | 3 | 3 | 3 | | |
| EHL0850 | HLJ II | Kuyuku 131 | Muling | 2007 | A | 3 | 3 | 3 | | ✓ |
| EHL0856 | HLJ II | Puyou 9 | Muling | 2007 | A | 3 | 3 | 3 | | |
| EHL0858 | HLJ II | Puyou 9 | Muling | 2007 | A | 3 | 3 | 3 | | |
| EHL0865 | HLJ III | Longjing 17 | Hulin | 2007 | A | 3 | 3 | 3 | | |
| EHL0866 | HLJ III | Longjing 17 | Hulin | 2007 | A | 3 | 3 | 3 | | |
| EHL0868 | HLJ III | Kenjiandao 10 | Hulin | 2007 | A | 3 | 3 | 3 | | ✓ |
| EHL0869 | HLJ III | Kenjiandao 10 | Hulin | 2007 | A | 3 | 3 | 3 | | ✓ |

| | | | | | | | | | |
|---|---|---|---|---|---|---|---|---|---|
| EHL0870 | HLJ III | Longjing 17 | Hulin | 2007 | A | 3 | 3 | 3 | |
| EHL0871 | HLJ III | Longjing 17 | Hulin | 2007 | A | 3 | 3 | 3 | |
| Barley and weed derived isolates | | | | | | | | | 5 |
| CHL1 | N/A | Barley | Smoeng, Thailand | 1998 | | 3 | 3 | 3 | ✓ |
| CHL2 | N/A | Barley | Smoeng, Thailand | 1998 | | 3 | 3 | 3 | ✓ |
| CHL27 | N/A | Weed | Smoeng, Thailand | 1998 | | 3 | 3 | 3 | ✓ |
| CHL30 | N/A | Weed | Smoeng, Thailand | 1998 | | 3 | 3 | 3 | |
| CHL31 | N/A | Weed | Smoeng, Thailand | 1998 | | 3 | 3 | 3 | |
| CHL32 | N/A | Weed | Smoeng, Thailand | 1998 | | 3 | 3 | 3 | |
| CHL325 | N/A | Weed | Guangzhou, China | 2001 | | 3 | 3 | 3 | |
| CHL326 | N/A | Weed | Guangzhou, China | 2001 | | 3 | 3 | 3 | |
| CHL329 | N/A | Weed | Guangzhou, China | 2001 | | 3 | 3 | 3 | ✓ |
| CHL330 | N/A | Weed | Guangzhou, China | 2001 | | 3 | 3 | 3 | ✓ |
| CHL331 | N/A | Weed | Guangzhou, China | 2001 | | 0 | 3 | 3 | |
| CHL332 | N/A | Weed | Guangzhou, China | 2001 | | 0 | 3 | 3 | |

[a] The rice-growing areas in the respective provinces, China, were adopted from Regionalization of Rice Cropping in China Edited in 1988 by China National Rice Research Institute, Hangzhou, China. Abbreviations GD I and GD II mean #1 and #2 rice-growing areas, respectively, in Guangdong province and so on.

[b] 2008E and 2008L mean Early season (from March to July) and Late season (from July to November) in 2008, respectively and so on.

[c] PCR product-based genotypes 0, 1, 2, 3, and 4, respectively, indicate none, highest, second higher, normal, and double binds of *AvrPib* products (also see Fig. 2).

[d] $D^{37\text{-}38}$, deletion at A37T38; A137G, SNP at #137 position and so on.

[e] Isolates being phenotypes/genotypes of both the major ones (V/1 and A/3) were subjected to selective resequencing, those being other three ones (V/2, V/3, and V/4) were all subjected to resequencing. A, avirulent; V, virulent, MV, moderately virulent.
